# DNA methylation in tumor-adjacent normal tissue in the course of head and neck squamous cell carcinoma as an epigenetic field for cancerization

**DOI:** 10.1186/1897-4287-10-S3-A20

**Published:** 2012-04-20

**Authors:** Joanna Katarzyna Strzelczyk, Łukasz Krakowczyk, Anna Płachetka, Grażyna Izdebska-Straszak, Andrzej Wiczkowski

**Affiliations:** 1 Chair and Department of General Biology, Medical University of Silesia, Zabrze, Poland; 2 Clinic of Oncological and Reconstructive Surgery, The Maria Sklodowska–Curie Memorial Cancer Center and Institute of Oncology, Gliwice, Poland

## 

DNA methylation is an epigenetic alteration, involved in human cancers. “Field cancerization”, also known as “field defect” in cancerogenesis has been explained by the presence of cells with genetic and epigenetic alterations, which are predisposed to cancer development. DNA methylation has been proposed as a candidate mediator of this field cancerization.

The aim of the study was to assess the methylation status of 6 genes promoter (MGMT, APC, TIMP3, RAR, ECAD and p16) in the tumor and in the adjacent normal tissue in patients suffering from head and neck squamous cell carcinoma (HNSCC).

The samples of tumor and adjacent normal tissue were obtained during surgery from 55 patients hospitalized at Maria Skłodowska-Curie Cancer Center and Institute of Oncology, Gliwice. After DNA isolation the methylation-specific polymerase chain reaction (MSP) was used to analyze the methylation status of the gene promoter mentioned above. Conventional histopathological parameters were assessed to prove the presence of squamous cell carcinoma while no neoplastic changes were detected histopathologically in the adjacent normal tissue. In order to establish the clinical relevance of methylation in HNSCC, correlation between methylation frequency of these genes promoter as well as the clinical stage of tumor, the patient’s age and gender were also considered. Ethics approval for this study was received from the Maria Skłodowska-Curie Cancer Center and Institute of Oncology Ethics Committee (No. KB/493-31/08).

Methylation of MGMT, APC, TIMP3, RAR, ECAD and p16 were detected in 38%, 33%, 61%, 67%, 29%, 49% of tumors, respectively. Some of the patients who delivered methylation–positive tumor samples, had also methylation–positive adjacent normal tissues. The genes promoter methylation were present in 20%, 31%, 39%, 49%, 11%, 42% of the tumor-adjacent normal tissues, respectively.

To sum up, the epigenetic modifications represented by DNA methylation in the adjacent normal tissue in the course of HNSCC point to the usefulness of DNA methylation as a marker of epigenetic cancerization field and of the future risk of cancer development.

